# Estrogen receptor β promotes bladder cancer growth and invasion *via* alteration of miR-92a/DAB2IP signals

**DOI:** 10.1038/s12276-018-0155-5

**Published:** 2018-11-20

**Authors:** Zhenyu Ou, Yongjie Wang, Jinbo Chen, Le Tao, Li Zuo, Deepak Sahasrabudhe, Jean Joseph, Long Wang, Shuyuan Yeh

**Affiliations:** 1 Departments of Urology and Plastic Surgery, Xiangya Hospital, Central South University, Changsha, 410008 China; 2 Departments of Urology and Pathology, University of Rochester Medical Center, Rochester, New York 14642 USA; 3 Department of Urology, Changzhou No. 2 People’s Hospital Affiliated to Nanjing Medical University, Changzhou, 213003 China; 4 Departments of Medicine, University of Rochester Medical Center, Rochester, New York 14642 USA

## Abstract

Although early studies suggested that bladder cancer (BCa) is more prevalent in men than in women, muscle-invasive rates are higher in women than in men, suggesting that sex hormones might play important roles in different stages of BCa progression. In this work, we found that estrogen receptor beta (ERβ) could increase BCa cell proliferation and invasion *via* alteration of miR-92a-mediated DAB2IP (DOC-2⁄DAB2 interacting protein) signals and that blocking miR-92a expression with an inhibitor could partially reverse ERβ-enhanced BCa cell growth and invasion. Further mechanism dissection found that ERβ could increase miR-92a expression at the transcriptional level *via* binding to the estrogen-response-element (ERE) on the 5′ promoter region of its host gene C13orf25. The ERβ up-regulated miR-92a could decrease DAB2IP tumor suppressor expression *via* binding to the miR-92a binding site located on the DAB2IP 3′ UTR. Preclinical studies using an in vivo mouse model also confirmed that targeting this newly identified ERβ/miR-92a/DAB2IP signal pathway with small molecules could suppress BCa progression. Together, these results might aid in the development of new therapies *via* targeting of this ERβ-mediated signal pathway to better suppress BCa progression.

## Bladder cancer: stopping the spread

Blocking the effects of the female hormone estrogen may increase the survival rate of women with bladder cancer (BCa). Although BCa is more common in men, tumors are more likely to invade neighboring tissues in women. Sex hormones and their receptors, which are known to affect progression of other cancers, may play a key role. A team led by Shuyuan Yeh at the University of Rochester Medical Center, USA, and Wang Long at Central South University, Changsha, China, investigated the role that estrogen receptor beta (ERβ) plays in BCa. They found that reducing ERβ levels made BCa cells less invasive. Further investigation revealed a way to block ERβ signaling, which made BCa tumors less likely to invade neighboring tissue in a mouse model. Understanding the ERβ signaling pathway may help to develop better treatments for BCa.

## Introduction

Bladder cancer (BCa) is a urological malignancy that has the highest lifetime treatment cost per patient among all types of solid cancers^1^. The incidence of BCa is nearly 3-fold higher in men than in women^2^. In contrast, the survival rate in female BCa patients is less than that in male BCa patients^3^. These results suggest that sex hormones, including androgen, androgen receptor, estrogens, and estrogen receptors (ERs), might contribute to this gender difference in BCa progression. The two major types of ERs are ER-alpha (ERα) and ER-beta (ERβ). Results from gene knockout mouse models showed that ERα has a protective role in inhibiting BCa initiation and growth, and ERβ promotes BCa cell growth and invasion^4,5^. However, the underlying mechanisms by which ERβ promotes BCa progression remain largely unclear. The microRNAs (miRNAs, miRs) are small highly conserved noncoding RNAs that can post-transcriptionally regulate target genes *via* binding to the 3′ untranslated region (3′UTR) of mRNAs^6^. Accumulating evidence indicates that miRNAs play important roles in tumor progression and in BCa growth and invasion^7,8^. The miRNAs can act as oncogenes to promote tumor development or as tumor suppressors to inhibit cancer development^9^. ERβ has been reported to directly regulate several miRNAs and affect tumor progression in different organs or tumors^10^. However, whether ERβ can promote BCa *via* regulation of miRNAs has not been fully investigated.

DOC-2/DAB-2 interacting protein (DAB2IP) belongs to the Ras GTPase-activating protein family and functions as a tumor suppressor to mediate tumor growth and invasion^11^. Clinical data indicated that DAB2IP was down-regulated in different human cancers, including prostate, lung, liver, and bladder^12–15^. In BCa, a low expression of DAB2IP was found to be associated with aggressive clinical features and worse outcomes^16^.

In this work, we introduce a previously unexplored mechanism by which ERβ modulates miR-92a/DAB2IP signals to promote BCa cell growth and invasion.

## Materials and methods

### Cell lines

Human BCa cell lines UMUC3 and J82 were purchased from the American type culture collection (ATCC, Ma-nas-sas, VA) and cultured in Dulbecco’s modified eagle media (DMEM) supplemented with 10% FBS, 2 mM L-glutamine, 100 IU/mL penicillin, 50 μg/mL streptomycin and maintained in a humidified 5% CO_2_ environment at 37 °C.

### Cell growth assay

Different BCa cells (at 5 × 10^3^) were plated into each well of 24-well plates. Viable cells were quantified at days 0, 2, 4, and 6 by incubation of cells in 0.5 mg/ml of 3-(4,5-dimethylthiazol-2-yl)-2,5-diphenyltetrazolium bromide (MTT) (Sigma-Aldrich, St. Louis, MO, USA) for 1 h and dissolution with DMSO. The absorbance was measured at a wavelength of 570 nm and data were presented as relative changes (fold).

### Transwell invasion assays

Invasion assays were performed using transwells with 8 μm pore-size inserts (Corning Inc., Corning, NY). The upper chamber inserts were coated with diluted Matrigel (BD Biosciences, Sparks, MD) for invasion assays. Amounts of 5 × 10^4^ BCa cells (in serum-free media) and 10% serum-containing media were plated in the upper and lower chambers, respectively. After a 24-hr incubation, the cells that invaded to the bottom sides of the transwell membranes were fixed and stained with 0.1% crystal violet. Positively stained cells were counted from six random fields. Quantitation is expressed as mean ± SD of triplicate repeats.

### 3D invasion assay

The 3D invasion assay was conducted according to a previous study^17^. In brief, Matrigel was thawed on ice, added to each well of 8-well glass chamber slides (at 50 μl/cm^2^) and spread evenly. Amounts of 1 × 10^5^ J82 cells were placed onto each well. Approximately 10 days later, the BCa cells were observed to form acini-like structures.

### RNA extraction and real-time quantitative PCR (q-PCR) analysis

Total RNAs were isolated using TRIzol reagent (Invitrogen, Grand Island, NY), and 2 µg was applied for reverse transcription using Superscript III transcriptase (Invitrogen). Quantitative real-time PCR (qRT-PCR) was conducted using a Bio-Rad CFX96 system with SYBR green to measure the mRNA expression level of the gene of interest. Expression levels were normalized to the expression of GAPDH mRNA.

### Western blot analysis

Western blot analysis was performed as previously described^4,5^. In brief, cells were washed with PBS and lysed in RIPA buffer. Quantified proteins were separated using 10% sodium dodecyl sulfate polyacrylamide gel electrophoresis and transferred onto PVDF membranes. After blocking with non-fat milk, the membranes were incubated with appropriate dilutions of specific primary antibodies. After washing in Tris-buffered saline plus 0.05% Tween-20, the blots were incubated with peroxidase-conjugated secondary antibodies and visualized using an enhanced chemiluminescence system (Thermo Fisher Scientific, Waltham, MA).

### Lentivirus packaging and transfection

ERβ shRNA was cloned into the lentiviral vector pLKO.1. To express ERβ, the cDNA of ERβ was cloned into the pWPI vector. Subsequently, 293 T cells were transfected with lentiviral constructs, packaging, and envelope plasmids (psPAX2 and pMD2.G) to produce the lentivirus. After 48 h, the lentivirus soup was harvested for immediate use or frozen for later use. The collected viruses were added to transduce the target cells for 24 hr. Cell cultures were refreshed with culture media and cultured for another three days to allow change of target protein expression.

### DAB2IP 3′UTR luciferase reporter assay

Promoters of the miR-92a host gene were obtained from genomic DNA analysis of HEK293T cells using Phusion High-Fidelity DNA Polymerase (NEB, Ipswich, MA) and constructed into a pGL3-basic vector (Promega, Madison, WI) using the Gibson assembly method. Fragments of DAB2IP 3′UTR containing wild-type or mutant miRNA-binding sites were cloned into the psiCheck2 construct (Promega) downstream of the Renilla luciferase ORF. Cells were plated in 24-well plates and transfected with cDNA using Lipofectamine 3000 (Invitrogen). The thymidine kinase promoter-Renilla luciferase reporter plasmid (pRL-TK) was used as the transfection efficiency control. Luciferase activity was measured *via* the Dual-Luciferase Assay (Promega) according to the manufacturer’s manual.

### Immunohistochemistry

Mouse bladder tissues were fixed in 10% (vol/vol) natural buffered formalin and embedded in paraffin. The embedded tissues were cut into sections with 5 μm thickness. The tissue sections were deparaffinized in xylene solution and rehydrated using gradient ethanol concentrations. Immunostaining was performed as described previously^17^.

### In vivo mouse BCa studies

Female nude mice 6–7 weeks of age were purchased from NCI. J82 cells were stably transfected with luciferase reporter gene (pcDNA3.0-luciferase) for monitoring of tumor growth and metastasis *via* the real-time in vivo imaging system (IVIS). Orthotopic xenografts of these BCa cells were applied according to our previous study^17^. In brief, after the female mice (7–8 weeks of age) were anesthetized, a lower mid-line abdominal incision was made, and the bladder was exposed. Amounts of 1 × 10^6^ J82 cells of different groups were resuspended in PBS and mixed with Matrigel (1:1). The cells were orthotopically injected into the muscle layer of bladder wall. Five weeks after tumor cell implantation, mice were injected with 150 mg/kg luciferin, and the fluorescent Imager (IVIS Spectrum, Caliper Life Sciences, Hopkinton, MA) was applied to monitor tumor growth and metastasis in live mice weekly. After eight weeks of tumor cell implantation, the mice were sacrificed, and the primary and metastatic tumors were further examined.

### Statistical analysis

All data are presented as the mean ± SD from at least three independent experiments. Statistical analyses were performed with SPSS 17.0 (SPSS Inc., Chicago, IL). Differences between two groups were analyzed using the unpaired Student’s *t*-test. *P* < 0.05 was considered statistically significant.

## Results

### ERβ increases BCa cell proliferation and invasion

**Fig. 1:**
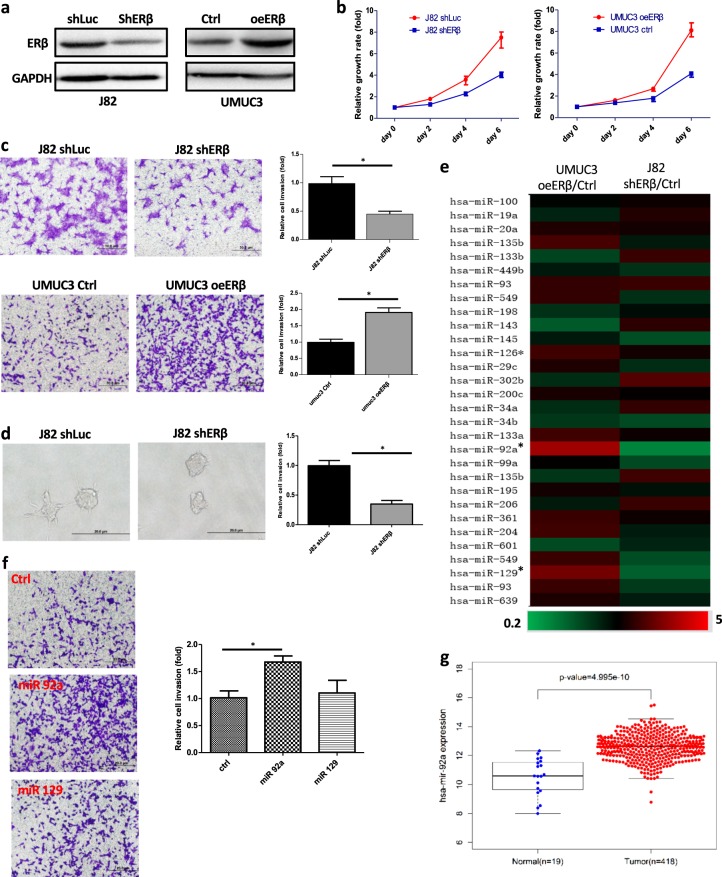
Identification of miRNA-92a as the downstream effector of ERβ in BCa. UMUC3 cells were transduced by lentivirus to overexpress ERβ (oeERβ) or vector control (Crtl). J82 cells were transduced with lentiviral-shERβ to knock down ERβ, or -shLuc as control for the following experiments. **a** Cell lysates were collected from lentivirus-transduced J82 and UMUC3 cells. Western blot analysis was performed, and proteins were detected by antibody against ERβ. **b** The MTT assay was used to analyze cell growth in J82 and UMUC3 cells transduced as described above to overexpress or knock down ERβ. **c** The Matrigel invasion assays were performed in J82 and UMUC3 cells transduced as described above. **d** 3D invasion assay showed that more protruding structures formed in control J82 cells than in J82 shERβ cells. **e** Real-time PCR screening of ERβ-regulated miRNAs related to BCa progression. We compared the PCR profile of miRNAs related to cancer cell growth and invasion in UMUC3 cells with overexpressed ERβ vs. vector (Ctrl) and J82 cells with shERβ vs. shLuc control (Ctrl). **f** Among the miRNAs screened, miR-92a and miR-129 expression were the most up-regulated by ERβ. We transduced lentiviral-miR-92a and lentiviral miR-129 into BCa cells, and the results showed that the increased miR-92a, not miR-129, can selectively increase BCa invasion. **g** We used the TCGA database to analyze the BCa sample array with miR-92a expression, and the results showed that BCa tumors have higher miR-92a expression than normal bladder tissues. In **c**, **d**, and **f**, data are presented as the mean ± SD. *p < 0.05

To test the ERβ impacts on BCa cell growth and invasion, we first transfected ERβ-cDNA or ERβ-shRNA into BCa UMUC3 and J82 cells, respectively, using the lentivirus system (Fig. 1a) and applied the MTT growth assay to study their effects. The results revealed that knockdown of ERβ with ERβ-shRNA in BCa J82 cells could decrease BCa cell growth, and ectopic expression of ERβ with ERβ-cDNA in UMUC3 cells could increase BCa cell growth, suggesting that ERβ might promote BCa cell growth (Fig. 1b).

To further examine the ERβ effects on BCa cell invasion, we applied the Matrigel invasion assay, and the results revealed that knockdown of ERβ in J82 cells decreased BCa cell invasion and that overexpression of ERβ in BCa UMUC3 cells increased BCa cell invasion (Fig. 1c). Similar results were also obtained when we replaced the Matrigel invasion assay with a 3D invasion assay showing that knockdown of ERβ in J82 cells significantly decreased cell invasion (Fig. 1d).

Taken together, the results from experiments using different BCa cell lines and assays all demonstrate that ERβ can increase BCa cell proliferation and invasion.

### ERβ increases BCa cell proliferation and invasion *via* an increased miR-92a expression

To dissect the molecular mechanism of how ERβ increases BCa cell proliferation and invasion, we focused on miRNAs as a growing body of evidence indicates that miRNAs could play important roles in tumor progression^18,19^. We applied the qRT-PCR assay to screen 30 BCa progression-related miRNAs after transduction of lentiviral ERβ-shRNA (shERβ) in J82 cells or ERβ-cDNA in UMUC3 cells. The results revealed that the expression of miR-92a and miR-129 were the most selectively altered after modulation of ERβ (Fig. 1e).

We examined the miR-92a and miR-129 effects on BCa cell invasion, and the results revealed that transduction of BCa with lentiviral miR-92a, but not miR-129, increased BCa cell invasion (Fig. 1f). Importantly, the results from The Cancer Genome Atlas (TCGA) databases further revealed that BCa has higher miR-92a expression than normal bladder tissues (Fig. 1g), suggesting miR-92a might play positive roles in promotion of BCa progression.

**Fig. 2:**
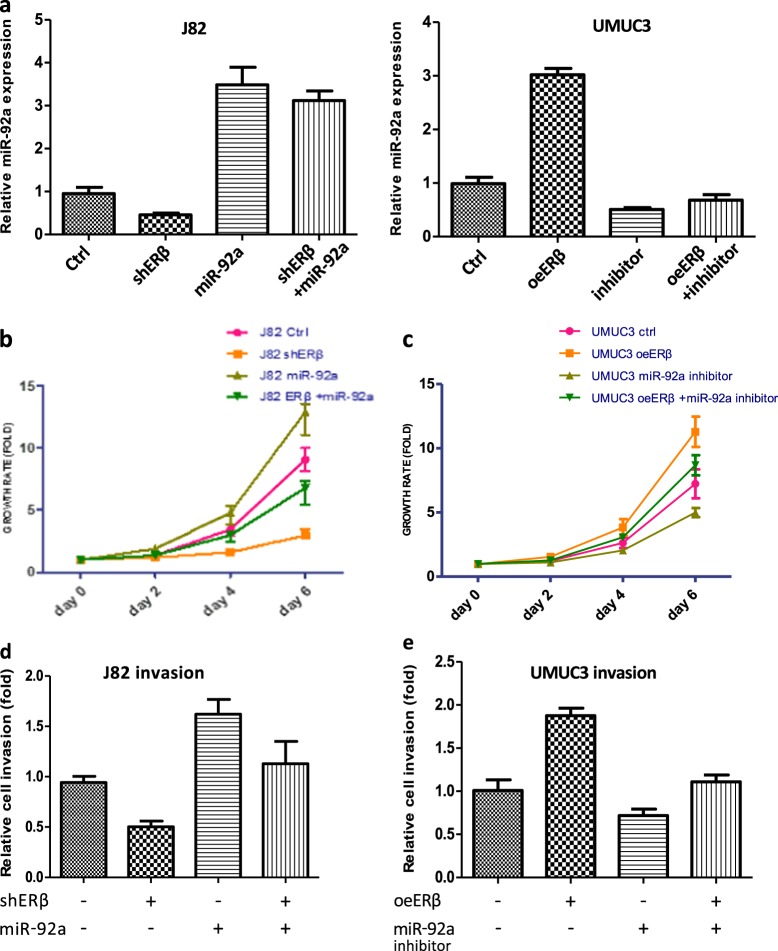
The miR-92a is an oncogenic miRNA that plays critical roles in mediating ERβ-promoted BCa cell growth and invasion. **a** Real-time PCR shows miR-92a levels in J82 cells transduced with lentiviral ERβ shRNA (shERβ) with/without miR-92a or control (Ctrl) and in UMUC3 cells overexpressed with lentiviral ERβ cDNA (oeERβ) with/without miR-92a inhibitor. We first showed in the J82 cells (**left**) that shERβ can reduce miR-92a expression. In UMUC3 cells (**right**), when ERβ is overexpressed, the miR-92a was up-regulated. The transfection of miR-92a inhibitor (oligonucleotide) can significantly reduce ERβ-upregulated miR-92 in UMUC3 cells. **b** J82 cells were prepared as in A for MTT assays. Ectopic miR-92a expression could reverse the shERβ-reduced BCa growth in J82 cells. Analysis of cell growth was conducted by MTT assay on days 0, 2, 4 and 6. **c** UMUC3 cells were prepared as described in A. The miRNA-92a inhibitor could dramatically reduce ERβ-promoted cell growth in these UMUC3 cells. **d** Ectopic miR-92a expression could reverse shERβ-reduced BCa invasion in J82 cells. Invasion assays were conducted using the transwell Matrigel invasion assay. **e** Transfection of UMUC3 cells with miR-92a inhibitor can reduce ERβ-promoted cell invasion. Data are represented as the mean ± SD

We examined the miR-92a effects on the ERβ-regulated BCa progression. We altered the expression of ERβ and miR-92a in J82 and UMUC3 cells (Fig. 2a), and examined the miR-92a effects on the ERβ influences on BCa progression. The results revealed that knockdown of ERβ in J82 cells decreased J82 cell growth and invasion and that further ectopic expression of miR-92a could partially reverse ERβ-shRNA-inhibited BCa cell growth and invasion (Fig. 2b, d). In an opposite approach, we also found that addition of ERβ in UMUC3 cells (UMUC3-oeERβ) led to promote the UMUC3 cell growth and invasion, and then transfection with miR-92a inhibitor (oligonucleotide) can partially reverse oeERβ-increased cell growth and invasion in UMUC3 cells (Fig. 2c, e).

Taken together, the results (Fig. 1e–g and Fig. 2a–e) from 2 BCa cell lines using different approaches all suggest that ERβ could increase BCa cell proliferation and invasion *via* alteration of miR-92a expression.

### Mechanism dissection: The ERβ-upregulated miR-92a can promote BCa cell growth and invasion *via* down-regulation of DAB2IP

**Fig. 3:**
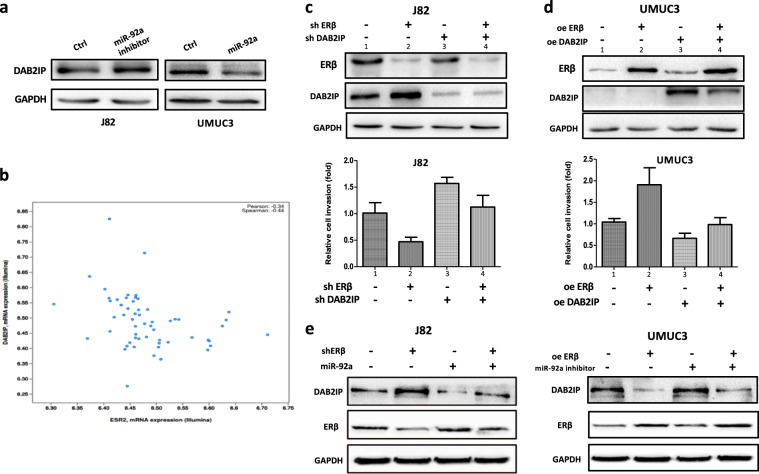
ERβ could up-regulate oncogenic miR-92a to down-regulate the tumor suppressor DAB2IP to promote the BCa cell invasion. **a** Western blot data showed DAP2IP protein expression is reversely regulated by miR-92a in J82 and UMUC3 cells with or without the miR-92a inhibitor or miR-92a, respectively. **b** Using cBioPortal databases to analyze the BCa tissues, results showed that DAB2IP and ERβ (ESR2) expression are inversely correlated. **c** Western blot (upper panel) and the transwell invasion assays (lower panel) after modulation of ERβ and DAB2IP using lentiviral transduction methods in J82 cells. Results showed shERβ (knockdown of ERβ) could up-regulate DAB2IP protein and inhibit cell invasion (lanes 2 vs 1). The addition of lentiviral shDAB2IP can reverse the shERβ-reduced J82 cell invasion (lanes 4 vs 2). **d** Western blot (upper panel) and the transwell invasion assays (lower panel) after modulation of ERβ and DAB2IP in UMUC3 cells. Over-expression of ERβ (oe ERβ) can down-regulate the DAB2IP protein expression and promote the UMUC3 invasion (lanes 2 vs 1). The ectopic DAP2IP over-expression (oeDAP2IP) can reverse the oeERβ up-regulated UMUC cell invasion (lanes 4 vs 3). As the oeDAP2IP can significantly increase the protein expression, the changes of endogenous DAP2IP protein bands in lanes 1 and 2 were barely observed. The oeERβ-down-regulated DAP2IP can be better observed in Fig. 3**e**, right panel. **e** Western blot analysis was used to assay ERβ and DAB2IP protein expressions in J82 (left panel) and UMUC3 (right panel) cells after modulation of the ERβ expressions (shERβ or oeERβ) with or without lentiviral miR-92a or miR-92a inhibitor. In **c** and **d**, quantifications of cell numbers that passed through the transwells were averaged from 5 representative fields. Data were presented as relative changes (fold).

To dissect the molecular mechanism(s) of how ERβ-upregulated miR-92a can promote BCa cell growth and invasion, we analyzed the miRNA prediction databases for its potential downstream target genes and identified the DAB2IP tumor suppressor as a potential target^20^. We applied Western blot analysis to confirm the protein expression and found that the addition of miR-92a could significantly decrease DAB2IP expression and that transfection of BCa cells with miR-92a inhibitor could increase DAB2IP expression (Fig. 3a). Importantly, a human clinical survey using the cBioPortal database^21^ also identified a negative correlation between the expressions of ERβ and DAB2IP (Fig. 3b).

We next applied the interruption approach to test the functional connections among ERβ, miR-92a, and DAP2IP in J82 and UMUC3 cells. The results with J82 cells revealed that knockdown of ERβ with ERβ-shRNA (shERβ) could increase DAB2IP expression and reduce J82 cell invasion (Fig. 3c, lanes 1 vs 2). Targeting of tumor suppressor DAB2IP with shRNA (shDAP2IP) can reduce the shERβ-increased DAP2IP protein (Fig. 3c, lanes 4 vs. 3, upper panel). Importantly, the addition of lentiviral shDAB2IP can effectively reverse the shERβ-reduced J82 cell invasion (Fig. 3c, lanes 2 vs 4, lower panel). As UMUC3 cells have a lower endogenous ERβ expression, we over-expression of ERβ (oeERβ) to examine the ERβ effects on the DAB2IP protein expression and UMUC3 cell invasion. Results showed that oeERβ can down-regulated DAB2IP and increase UMUC cell invasion (Fig 3d, lanes 1 vs 2, lower panel). Furthermore, the ectopic DAP2IP over-expression (oeDAP2IP) can reverse the oeERβ up-regulated UMUC cell invasion (Fig. 3d, lanes 2 vs 4, lower panel). As the oeDAP2IP can significantly increase the protein expression, the changes of endogenous DAP2IP protein bands in lanes 1 and 2 were barely observed due to the relative detection of protein amount difference (Fig. 3d, upper panel). Indeed, the oeERβ-down-regulated DAP2IP can be better observed in UMUC3 in the Fig. 3e (lanes 1 vs 2, right panel). Supportively, we found that ERβ-shRNA-increased DAB2IP expression could be significantly reversed after addition of miR-92a to J82 cells (Fig. 3e, left panel). As expected, oeERβ-decreased DAB2IP expression (Fig. 3e, right panel) could also be effectively reversed after transfection of UMUC3 cells with miR-92a inhibitor.

 Taken together, the results from clinical BCa tissues and 2 different BCa cell studies (Fig. 3a–e) consistently show that a functional connection exists among ERβ, miR-92a, and DAB2IP. The ERβ could up-regulate oncogenic miR-92a to down-regulate the tumor suppressor DAB2IP, and then increase BCa cell invasion.

### Mechanism dissection: ERβ/miR-92a can modulate DAB2IP expression *via* binding to the DAB2IP 3′-UTR

**Fig. 4:**
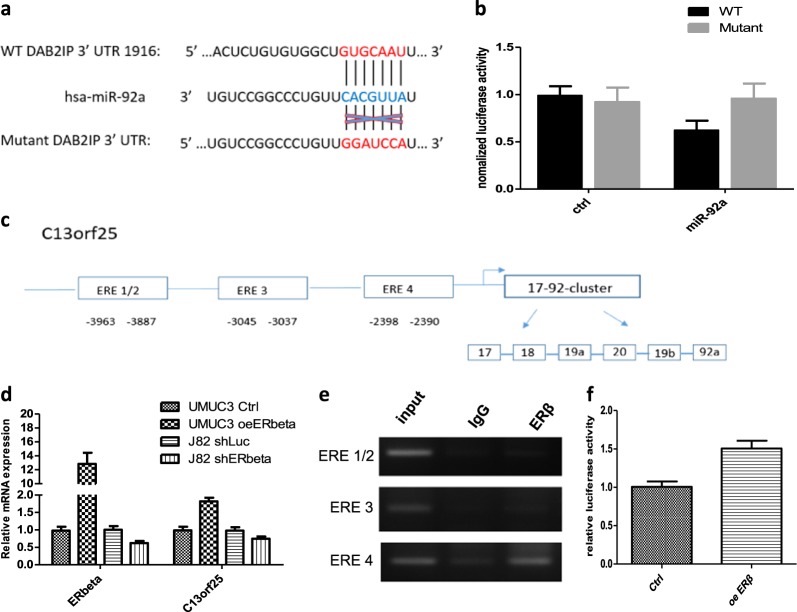
Mechanism dissection of how miR-92a inhibits DAB2IP expression and how ERβ transcriptionally up-regulates miR-92a expression. **a** DAB2IP 3′UTR containing wild-type (WT) or mutant miRNA-binding sites were cloned into the psiCheck2 vector. **b** We co-transfected miR-92a and psiCheck2-DAB2IP 3′UTR constructs containing WT or mutant miR92a-targeted binding regions into 293 T cells, and the luciferase activity was assayed. **c** Schematic depiction of the potential ERβ-binding sites on the 5′ promoter region of the C13orf25 gene that could produce the miR-92a. **d** Expression of C13orf25 in UMUC3 cells with/without oeERβ and J82 cells with/without shERβ were detected by real-time qPCR. **e** The ChIP assay showed that ERβ could bind to the 4^th^ predicted ERE site in the promoter of the miR-92a host gene. **f** UMUC3 cells were transfected with a 2.4 kb promoter-pGL3 luciferase reporter plasmid for luciferase activity assay. The Renilla luciferase reporter was used as an internal control for transfection efficiency. Each experiment was performed independently 3 times. In **b**, **d**, and **f**, data are presented as the mean ± SD. *P < 0.05

To further dissect the molecular mechanism by which miR-92a regulates DAB2IP expression, we tested whether miR-92a can potentially bind to the DAB2IP 3′UTR to regulate the protein expression of DAB2IP. Based on miRNA database analysis, we found a potential miR92a binding site on the DAB2IP 3′UTR (Fig. 4a). We constructed (length = 2 kb) a wild-type (WT) 3′UTR of DAB2IP on the 3′ end of luciferase cDNA (psiCheck2–DAB2IP 3′UTR-WT), and the luciferase reporter assay showed that miR-92a could decrease the luciferase activity of psiCheck2–DAB2IP 3′UTR-WT (Fig. 4b, **lane 3 vs. 1**). To determine whether this binding of miR-92a to the DAB2IP 3′UTR is specific, we also constructed a mutant 3′UTR of DAB2IP, with a mutation that destroys the miR92a binding site (psiCheck2–DAB2IP 3′UTR-mt). The luciferase activity assay results showed that this mutation could prevent the binding of miR-92a (Fig. 4b, **lane 4 vs. 2**).

### Mechanism dissection: How ERβ can modulate miR-92a at the transcriptional level

Because the mature form of miR-92a was derived from its host gene C13orf25 (Fig. 4c), we also tested whether ERβ could transcriptionally regulate the expression of C13orf25. Real-time quantitative-PCR showed that enforced expression of ERβ could up-regulate C13orf25 and that knockdown of ERβ could down-regulate C13orf25 at the mRNA levels (Fig. 4d).

To investigate whether ERβ could regulate C13orf25 by transcriptional regulation of its promoter activity, we analyzed the potential ERβ-response-elements (EREs) on the 5′ promoter of the C13orf25 gene. The bioinformatics analysis showed four potential EREs in the 4 kb promoter region (Fig. 4c), namely, locations (#1 and #2) −3963 to −3887 bp, (#3) −3045 to −3035 bp, and (#4) −2398 to −2390 bp. To test the binding of ERβ to these potential EREs, the chromosome immuno-precipitation assay (ChIP) was applied, and the results showed that ERβ could selectively bind to one of the candidate ERE sites, i.e., the predicted #4 ERE, located from the −2398 base to the −2390 base (Fig. 4e**)**. We cloned 2.4 kb of the promoter region into the pGL3 vector for the luciferase reporter assay. The results showed that ERβ could increase the C13orf25 (2.4 kb) promoter-luciferase reporter (2.4 kb) activity **(**Fig. 4f).

### Preclinical study using an in vivo mouse BCa model to demonstrate that ERβ increases BCa metastasis *via* alteration of miR-92a/DAB2IP signals

To confirm the in vitro results in an in vivo mouse BCa model, we used J82 cells orthotopically xenografted into the bladder walls of female nude mice. The implanted J82 cells were stably transfected with pCDNA3-luciferase (J82-Luc) for monitoring of the tumor growth and metastasis *via* the real-time in vivo imaging system (IVIS). The J82-Luc cells were transduced with lentiviral vector control, shERβ, miR-92a, or shERβ + miR-92a, and 1 × 10^6^ of each type of cells were implanted into the bladder wall of 6-week-old female nude mice (*n* = 8). 5 weeks after tumor implantation, we applied IVIS to monitor tumor development weekly for an additional 3 weeks.

**Fig. 5:**
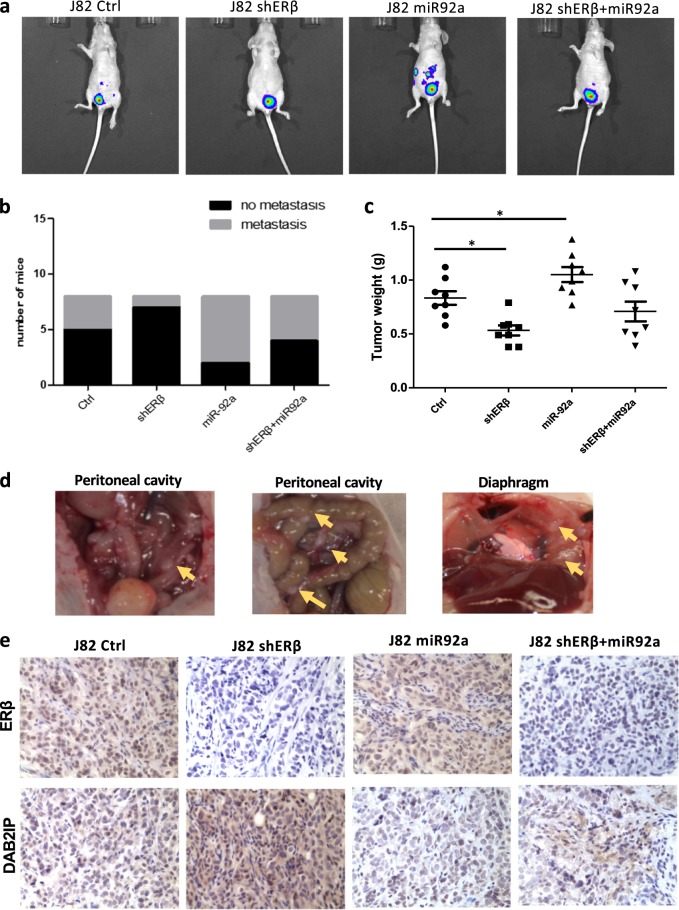
ERβ promotes metastasis of BCa *via* up-regulation of miR-92a and down-regulation of DAB2IP in the in vivo mouse BCa model. The implanted J82 cells were stably transfected with pCDNA3-luciferase (J82-Luc) for monitoring of tumor growth and metastasis *via* the real-time in vivo imaging system (IVIS). **a** Representative IVIS images of the four groups of mice showed the outcome at eight weeks after orthotopical implantation of BCa J82-luc cells into the bladder wall of female nude mice. **b** Incidence of metastases in different groups of mice at eight weeks after BCa cell implantation. **c** Comparison of bladder weights of different mouse tumor groups. **d** The images show representative metastatic lymph foci (yellow arrows) around the peritoneal and diaphragm regions. **e** Representative images of IHC results for ERβ and DAB2IP in four groups of orthotopic bladder tumor tissues (400 × ). In **c**, data are presented as the mean ± SD, *P < 0.05

The mice in all four groups developed tumors with different growth and metastasis rates. The IVIS imaging results revealed that the mice implanted with J82-Luc cells with miR-92a overexpression had the highest metastatic incidence (75%). In contrast, the J82-luc shERβ tumor group had the lowest metastatic incidence (12.5%). Comparison of female mice with J82-luc + shERβ *vs*. those with J82-luc + shERβ + miR-92a xenografts showed that miR-92a can reverse the shERβ-reduced metastatic foci formation (Fig. 5a, b).

After the mice were euthanized, we examined the weights of bladders with tumors and the total metastatic foci. The average bladder weight of the J82 shERβ group was significantly lower than that of the control group, and the average bladder weight of the miR-92a group was higher than that of the control group (Fig. 5c). The results revealed that the metastatic foci were located in the retroperitoneum, peritoneal, and diaphragm regions (Fig. 5d). The results from IHC staining in Fig. 5e also indicated the correlated expressional changes of DAB2IP by ERβ in the bladder tumors, which were consistent with the in vitro data. We used qRT-PCR to examine the expression levels of ERβ mRNA、miRNA-92a and DAB2IP mRNA in the metastatic foci collected from peritoneal regions. As shown in the Supplementary Figure 1: The miRNA-92a was up-regulated in the metastatic foci in J82 miRNA-92a group compared to other groups and the miRNA-92a was down-regulated in J82 shERβ group. The DAB2IP mRNA did not significantly change among those four tumor groups which implied that DAB2IP may be regulated by miRNA-92a through post-transcriptional control. These in vivo findings are consistent with the in vitro results. Together, our results demonstrated that ERβ could increase BCa growth and metastasis *via* miR-92a/DAB2IP signals in the orthotopically implanted mouse BCa model.

## Discussion

Clinical data show that sexual disparity exists in BCa incidence and progression. Men are more likely to get BCa, and in contrast, women have a higher muscle-invasive BCa rate than men. ERs might be responsible for this sexual difference. In the context of BCa, the two receptors ERα and ERβ have been studied^3,22,23^. Observations in clinical samples implicated that ERβ expression is positively linked to cancer stage^23^. Our previous studies using ER knockout mouse models showed that ERα can inhibit BCa initiation and growth^4^ and that ERβ can promote BCa growth and invasion^5^. We applied a BBN-induced mouse BCa model to demonstrate that knockout of ERβ can decrease BCa occurrence significantly^5^. Although our previous study showed that ERβ could function *via* up-regulation of MCM5^5^ CCL2^24^, and IL-1/c-Met^25^ pathways to promote the BCa, the mechanisms by which ERβ can increase BCa growth and metastasis are not yet fully investigated.

As an important player in promoting BCa progression, ERβ could have multiple ways to exert its function. We focused on miRNAs because they are involved in the progression of BCa^26,27^. In this work, we screened 30 miRNAs related to BCa progression^26,28^ and found that a subset of them are regulated by ERβ. Interestingly, we found miR-92a, which is located in a primary transcript known as C13orf25 and belongs to the miR-17–92 cluster^29^, is a critical downstream factor of ERβ. This miRNA has been reported to enhance the progression of different cancers, including lung cancer, hepatocellular carcinoma, colorectal cancer and neuroblastoma^30,31^. Thus far, several targeted genes of miR-92a have been identified, such as the Bcl-2-interacting mediator of cell death (BIM) and reversion-inducing cysteine-rich protein with Kazal motifs (RECK), which are all tumor suppressors^30^. In the current study, overexpression of miR-92a could significantly increase BCa cell growth and invasion. We supply the first evidence that miR-92a expression is regulated by ERβ, and exhibits oncogenic activity in BCa. Importantly, interruption of miR-92a can partially block ERβ’s function in BCa progression.

We also identified DAB2IP as a new in vivo target of miR-92a. DAB2IP is involved in the development of many cancers. By interacting with DIP1⁄2, a Ras GTPase-activating protein, DAB2IP can form a protein complex with a negative regulatory activity that modulates the Ras-mediated signaling pathway^32^. As a tumor suppressor, DAB2IP was found to be down-regulated in BCa tissues. Clinical sample analysis and functional experiments revealed that DAB2IP plays an important role in BCa progression and was strongly correlated with unfavorable patient outcomes^16^. Loss of DAB2IP can also influence the response of BCa cells to ionizing radiation^33^. In our study, DAB2IP was found to be down-regulated in BCa cells with ERβ overexpression and up-regulated in BCa cells with knockdown of endogenous ERβ (Fig. 2). Consistent results were obtained in an orthotopically implanted mouse BCa model (Fig. 5). Ectopic expression of DAB2IP can partially reverse ERβ overexpression-mediated BCa cell growth and invasion, suggesting that DAB2IP indeed contributes to ERβ’s function in BCa. The IHC results of the in vivo mouse BCa tissues also showed that ERβ could down-regulate DAB2IP expression (Fig. 5e).

By analyzing the cBioPortal databases, we found an inverse correlation exists between the expressions of ERβ and DAB2IP. These clinical data strongly support our cell line findings. In the current study, we found a new ERβ downstream miR-92a/DAB2IP signaling pathway that is critical for the ERβ function of promoting BCa progression. The control of growth and invasion of BCa cells by the ERβ/miR-92a/DAB2IP axis might offer new strategies for treatment of BCa. Future studies might allow the development of an effective therapeutic strategy to interrupt these newly identified signals and better suppress BCa progression.

## Electronic supplementary material

Supplementary Figure 1
