# Supplementary material for: Estrogen receptor β promotes bladder cancer growth and invasion via alteration of miR-92a/DAB2IP signals
Source: Exp Mol Med. 2018 Nov 20;50(11):152. doi: 10.1038/s12276-018-0155-5 (PMC6243995; doi:10.1038/s12276-018-0155-5)

Figure Legends

Supplementary Figure 1. QRT-PCR results showed the ERβ mRNA、miRNA-92a and DAB2IP mRNA expressing levels in the peritoneal metastatic foci in different groups of mice.

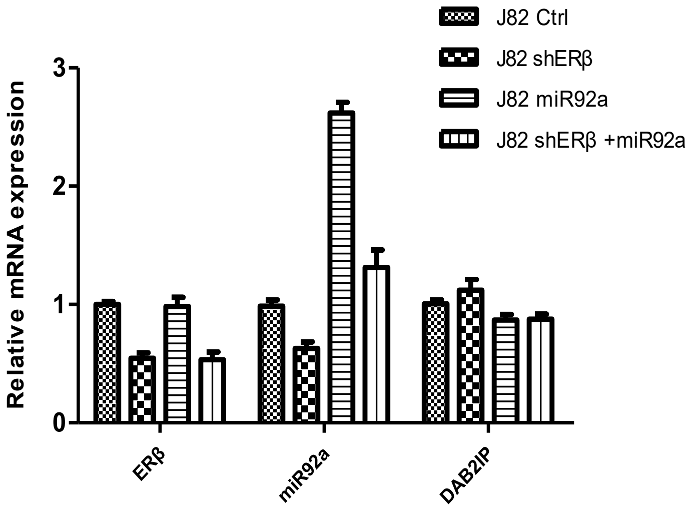

Supplement: Supplementary file 1 — Supplementary Figure 1 [file 12276_2018_155_MOESM1_ESM.pdf]
